# Large Granular Lymphocytic Leukemia: A Report of Response to Rituximab

**DOI:** 10.1155/2017/7506542

**Published:** 2017-07-18

**Authors:** Uroosa Ibrahim, Sara Parylo, Shiksha Kedia, Shafinaz Hussein, Jean Paul Atallah

**Affiliations:** ^1^Department of Hematology/Oncology, Staten Island University Hospital, 475 Seaview Avenue, Staten Island, NY 10305, USA; ^2^Department of Medicine, Staten Island University Hospital, 475 Seaview Avenue, Staten Island, NY 10305, USA; ^3^Department of Pathology, Staten Island University Hospital, 475 Seaview Avenue, Staten Island, NY 10305, USA

## Abstract

Large granular lymphocytic (LGL) leukemia is a rare form of low grade leukemia characterized by large cytotoxic T cells or natural killer cells on morphological examination. Immunosuppressive therapy is employed as first-line therapy. Treatment options in refractory cases include the anti-CD52 antibody alemtuzumab and purine analogues. We report a rare case that responded to the anti-CD20 monoclonal antibody rituximab. A 77-year-old female presented with complaints of fatigue, fever, and chills of 3 months' duration. A CBC showed that pancytopenia with an absolute neutrophil count (ANC) was 0. Peripheral blood flow cytometry detected increased number of T cell large granular lymphocytes and T cell receptor rearrangement study detected a clonal T cell population. Bone marrow biopsy showed peripheral T cell lymphoma, most consistent with T-large granulocytic leukemia. The patient was treated with prednisone and oral cyclophosphamide for four months with no response. Thereafter, she received four weekly infusions of rituximab with improvement in her blood counts. A response to rituximab in refractory cases such as ours has been reported and may guide us towards exploring other immune-based therapeutics in this rare disease.

## 1. Introduction

Large granular lymphocytic (LGL) leukemia is a rare form of low grade leukemia characterized by large cells on morphological examination. The LGL cells may be of the cytotoxic T cell or natural killer cell lineage. LGL leukemia may be associated with autoimmune disease or a hematological disorder such as monoclonal gammopathy of undetermined significance or myelodysplasia. The patients typically present with constitutional symptoms, cytopenias, and organomegaly. When treatment is indicated, immunosuppressive therapy such as methotrexate, cyclosporine, or cyclophosphamide is employed. Treatment options in refractory cases include the anti-CD52 antibody alemtuzumab and purine analogues. We report a rare case that responded to the anti-CD20 monoclonal antibody rituximab.

## 2. Case

A 77-year-old female presented to our hospital with complaints of worsening fatigue, subjective fever, and chills of 3 months' duration. Review of systems was pertinent for decreased appetite, mouth sores, and 20 pounds of weight loss over the last 6 months. Her past medical history included hypertension, atrial fibrillation, and hypothyroidism for which she was on metoprolol, warfarin, and levothyroxine, respectively. Her complete blood count showed a white count of 1.66 × 10^9^/L, hemoglobin of 8.6 gm/dL, and platelets of 122 × 10^9^/L. Absolute neutrophil count (ANC) was 0. A CBC from two years prior to presentation was normal. Physical examination revealed a palpable spleen 3 cm below the left costal margin. Laboratory work-up including hepatitis B, hepatitis C, and HIV serology, vitamin B12, folate, free light chains, and serum protein electrophoresis was normal. C-reactive protein and erythrocyte sedimentation rate (ESR) were 2.21 mg/dl and 122 mm/hr, respectively. Beta-2-microglobulin was elevated at 24.5 mg/L and creatinine at 2 mg/dL. Ferritin was 328 ng/mL.

**Figure 1:**
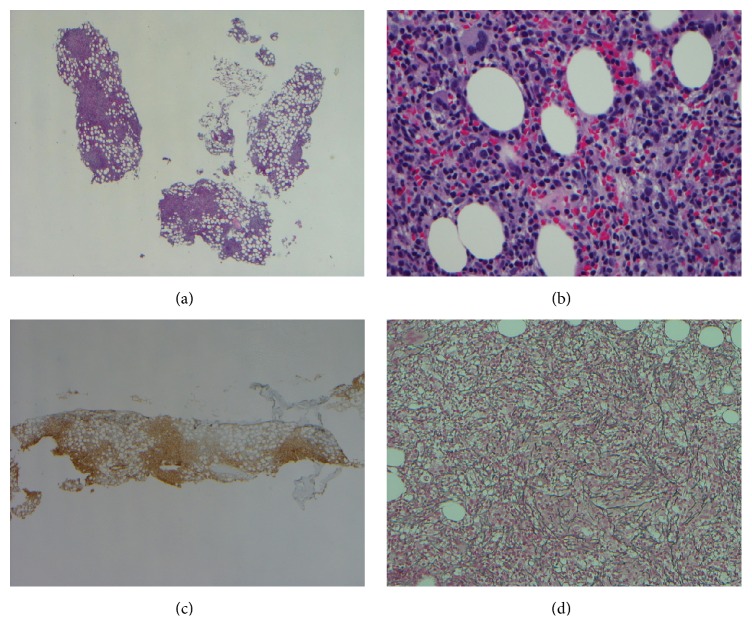
The bone marrow biopsy showed a nodular proliferation of T cells ((a) H&E, 20x; (b) H&E, 400x; (c) CD3, 20x) and increased reticulin fibrosis ((d) reticulin stain, 400x).

A Computed Tomography (CT) scan of the chest showed a 1.5 cm left subaortic lymph node and a 1.5 cm subcarinal lymph node with cardiomegaly and a small pericardial effusion. CT abdomen revealed an enlarged spleen measuring 13 × 6 × 11.5 cm. Flow cytometry of peripheral blood detected increased number of T cell large granular lymphocytes comprising 25% of the cells and expressing CD 2, 3, 5, 7, 8, and 57. T cell receptor (TCR) rearrangement studies detected a clonal T cell population. Bone marrow aspirate was unsuccessful due to a dry tap and the biopsy showed peripheral T cell lymphoma, most consistent with T-large granulocytic leukemia. Immunohistochemistry of lymphocytes showed T cells expressing pan T cell markers including CD2, CD3, CD5, and CD7 (Figure 1). Many B cells with CD79a and CD20 were seen. CD10 and CD56 were negative. CD57 showed positivity for many T cells and weak expression for perforin. Ki67 was <5% within the lymphoid aggregates. ALK and cyclin D 1 were negative. In situ hybridization for EBV RNA (EBER) was negative.

The patient was started on prednisone 40 mg and oral cyclophosphamide 50 mg daily. Renal insufficiency precluded the use of methotrexate and cyclosporine. The patient did not seem to respond and the dose of cyclophosphamide was increased to 100 mg. At four months, the patient was hospitalized for neutropenic fever. A decision was made to discontinue cyclophosphamide. Given the significant number of CD20 positive B cells on the bone marrow biopsy specimen, a decision to treat her with rituximab was made. After four weekly infusions of 375 mg/m^2^, her CBC improved to a WBC of 5 × 10^9^/L, ANC of 2430, hemoglobin of 10.4 gm/dL, and platelet count of 200 × 10^9^/L.

## 3. Discussion

Large granular lymphocytic (LGL) leukemia is a type of chronic leukemia comprising 2–5 percent of chronic lymphoproliferative diseases and has a median age of diagnosis of about 66.5 years [1]. The World Health Organization (WHO) classifies LGL disorders into three types as follows: T cell large granular lymphocytic leukemia (T-LGLL), which is the most common type, chronic lymphoproliferative disorders of NK cells (CLPD-NK) comprising about 5% of LGL leukemia, and aggressive NK cell leukemia accounting for about 10% of LGL leukemia [1, 2]. LGL cells are composed of CD3-NK cells and CD3+ T cells that are programmed to bind to infected target cells and undergo apoptosis. However, in patients with LGL leukemia, these cells continue to survive and proliferate [2].

A diagnosis of LGL leukemia requires a peripheral smear and bone marrow biopsy examination and flow cytometry to determine T cell versus NK cell lineage. A circulating LGL count of >0.4 or 0.5 g/L is also required for diagnosis [1]. Other causes, such as viral infections, hairy cell leukemia, autoimmune neutropenia, and myelodysplastic syndrome, must be ruled out [3].

Felty syndrome is classically defined as rheumatoid arthritis, neutropenia, and splenomegaly. There is considerable overlap between Felty syndrome and LGL leukemia. 11–36% of patients with LGL leukemia have been shown to have rheumatoid arthritis, and 30–40% of patients with Felty syndrome have been shown to have increased LGL counts [1], leading to the hypothesis that Felty syndrome and LGL leukemia with rheumatoid arthritis may share a similar etiology [2].

The most common presentation includes fever, night sweats, and unintentional weight loss. Laboratory work-up may reveal red cell aplasia, chronic neutropenia, and anemia, and a history of recurrent infections may be present. Whereas splenomegaly is common, hepatomegaly and lymphadenopathy are rare. About 20 percent of patients are diagnosed with autoimmune diseases, such as a rheumatoid arthritis, prior to the onset of LGL leukemia. For some asymptomatic patients, watchful waiting with periodic laboratory evaluations may be appropriate. However, patients with moderate to severe neutropenia, recurrent infections, symptomatic anemia, transfusion requirement, or concomitant autoimmune diseases, such as rheumatoid arthritis, require treatment [3, 4].

Treatment for LGL leukemia is not standardized. The most common documented initial treatments for T-LGLL and CLPD-NK include low-dose methotrexate, cyclophosphamide, and cyclosporine. Patients are typically treated for four months before a response assessment is made. Patients may be continued on methotrexate and cyclosporine indefinitely, as long as their disease responds to the therapy. Relapse is almost immediate upon treatment discontinuation [3–5]. In contrast, cyclophosphamide is typically given only for about 4 to 12 months due to risk of bladder toxicity and malignancy potential. Complete clinical response is defined as normalization of the platelet count, absolute neutrophil count, and hemoglobin and a lymphocyte count of less than 4 × 10^9^/L. Partial response is defined as improvement of blood counts but persistence of neutropenia [2, 5].

For refractory LGL leukemia, treatment options include purine analogs, such as fludarabine, the anti-CD52 monoclonal antibody alemtuzumab, or splenectomy [2]. Pelliccia et al. have reported a case of a 57-year-old male without known autoimmune disorders with refractory T-LGLL and multiple myeloma, treated with bortezomib and then subsequent lenalidomide, resulting in complete remission of T-LGLL and partial remission of multiple myeloma [6]. Raposo et al. described a case of a 67-year-old female with rheumatoid arthritis, with increased large granular lymphocyte cells on the peripheral blood smear treated sequentially with methotrexate, leflunomide, and etanercept, all stopped because of intolerable adverse effects. The patient was then treated with rituximab, with improvement of neutrophil count [7]. Cornec et al. presented two cases of long-term remission of T-LGLL associated with rheumatoid arthritis following treatment with rituximab. In the first case, the patient was treated with methotrexate, cyclosporine, and G-CSF (Granulocyte-Colony Stimulating Factor), which were discontinued secondary to adverse effects, followed by three infusions of rituximab, which led to complete remission of LGLL, which persisted for eight years at the time of publication. The second case was a male diagnosed with T cell LGLL, followed by a diagnosis of rheumatoid arthritis four months later. The patient was treated with methotrexate and prednisone without improvement. Two infusions of rituximab resulted in resolution of symptoms and normalization of the ANC, despite persistence of monoclonal T cells [8].

Aggressive LGL leukemia may be treated with CHOP-like chemotherapy (cyclophosphamide, adriamycin, vincristine, and prednisone) as well as allogenic stem cell transplantation but typically has a poor prognosis, with most patients dying within 1-2 months of presentation regardless of treatment rendered [2, 4]. For all types of LGL leukemia, medical comorbidities and age of 60 years or greater at time of diagnosis have been shown to independently predict poor survival [9].

## 4. Conclusion

LGL leukemia is largely treated with immunosuppression and no curative therapeutic modality is known to date. A response to rituximab in refractory cases such as ours has been reported and may guide us towards exploring other immune-based therapeutics in this rare disease.
